# Cognitive and Mood Profiles Among Patients With Stiff Person Syndrome Spectrum Disorders

**DOI:** 10.3389/fneur.2022.865462

**Published:** 2022-05-27

**Authors:** Carol K. Chan, Daniela A. Pimentel Maldonado, Yujie Wang, Danielle Obando, Abbey J. Hughes, Scott D. Newsome

**Affiliations:** ^1^Department of Psychiatry and Behavioral Sciences, Johns Hopkins University, Baltimore, MD, United States; ^2^Department of Neurology, Johns Hopkins University, Baltimore, MD, United States; ^3^Department of Neurology, University of Washington, Seattle, WA, United States; ^4^Department of Physical Medicine and Rehabilitation, Johns Hopkins University, Baltimore, MD, United States

**Keywords:** stiff person syndrome, cognition, attention, verbal fluency, depression, anxiety

## Abstract

An emerging body of evidence suggests that changes in cognitive and emotional function are common aspects of stiff person spectrum disorders (SPSD). We sought to examine the pattern of cognitive impairment and psychiatric symptoms in SPSD.

**Methods:**

A retrospective review of medical records was conducted for patients seen at the Johns Hopkins Stiff Person Syndrome (SPS) center from 1997 to January 1st, 2020. Individuals who had received formal cognitive testing as part of routine clinical care for patient-reported cognitive changes were included. Demographics, prevalence of cognitive impairment, psychoactive medication use, and clinically significant psychiatric symptoms were described.

**Results:**

Out of 205 patients screened, 20 completed cognitive testing (75% female, mean age 47.4 years). The most common domains of impairment were verbal learning and recall memory (*n* = 14, 70%), verbal fluency (*n* = 10, 50%), processing speed (*n* = 8, 40%), and attention (*n* = 8, 40%). 9/11 patients assessed for depression reported clinically significant symptoms, and 4/9 patients assessed for anxiety reported clinically significant symptoms.

**Conclusions:**

Screening for cognitive impairment in SPSD should utilize testing that assesses verbal learning and recall, phonemic verbal fluency, attention, and processing speed. Moreover, it is important to evaluate for co-existing depression and anxiety symptoms, as these are common in SPSD.

## Introduction

Stiff person spectrum disorders (SPSD) are immune-mediated disorders most often characterized by rigidity, unpredictable and painful spasms, and heightened sensitivity to external stimuli (1). Anti-glutamic acid decarboxylase 65 (anti-GAD65) antibodies are thought to play a role in the GABAergic dysfunction in SPSD. While it is classified as a neurologic disorder, research is limited regarding the effects of stiff person syndrome (SPS) on cognitive and emotional function (1, 2).

SPSD has been associated with lower than expected performance on cognitive testing relative to estimated premorbid intelligence (3). Furthermore, the presence of anti-GAD65 antibody has been associated with cognitive impairment in patients with neurological conditions (4), type 2 diabetes (5), and in animal models (6). In addition to cognitive dysfunction, patients with SPSD are also more likely than the general population to report anxiety and depressive symptoms, and to regularly use prescription benzodiazepines and muscle relaxants (7), all of which may contribute to poor performance on cognitive testing (8–10). To our knowledge, only two prior studies have assessed cognitive symptoms in patients with SPSD (3, 11). While one also included measures of psychiatric symptoms (3), neither study reported on psychiatric symptoms or patterns of medication use in the context of cognitive performance.

The aims of this case series were to: (1) describe the pattern of cognitive impairment in patients with SPSD who reported concerns of cognitive impairment and participated in cognitive testing as part of routine clinical care; and (2) examine the frequency of mood symptoms and use of benzodiazepines and muscle relaxants in the most commonly impaired cognitive domains.

## Methods

A retrospective review of medical records was conducted for patients seen at the Johns Hopkins SPS center from 1997 to January 1st, 2020. All patients had provided informed consent to participate in a longitudinal observational study of clinical characteristics in SPS, approved by the Johns Hopkins Institutional Review Board.

Medical records were reviewed for formal cognitive testing, performed by either a licensed psychologist or a speech and language pathologist, as part of routine clinical care for patient-reported cognitive changes. Information on demographics, clinical characteristics, medical comorbidities, and medications at the time of cognitive testing were extracted. Patients with limbic encephalitis, co-existing intractable epilepsy, and/or other neurological conditions known to affect cognitive performance (e.g., Alzheimer's disease, multiple sclerosis, etc.) were excluded.

As a retrospective review of cognitive testing performed as part of routine clinical care, cognitive testing batteries used were determined at the discretion of the provider and therefore not standardized. Details of cognitive testing reports were extracted; results were interpreted as “impaired” if records included descriptive labels of “abnormal”, “extremely low”, or “weak”. If no descriptive interpretation was offered, an adjusted percentile score of <2 or z-score of < −2 (e.g., more than 2 standard deviations below mean) was interpreted as “impaired” (12). If standardized instruments of psychological symptoms (e.g., depression and/or anxiety) were administered, the scores and descriptive labels (e.g., “clinically significant”) were extracted.

Demographic and clinical characteristics were evaluated using descriptive statistics, *t*-test for continuous variables and chi-squared test for dichotomous variables using R Studio Version 1.2.5033 (13). Significance was set at *p* < 0.05. Frequency of domain-specific cognitive impairment across individuals with cognitive testing was examined. For the 4 most commonly impaired cognitive domains, frequency of prescription antidepressants (e.g., selective serotonin reuptake inhibitors, serotonin and norepinephrine reuptake inhibitors), benzodiazepines (e.g., lorazepam, diazepam, clonazepam) and non-benzodiazepine muscle relaxants (e.g., cyclobenzaprine, baclofen, dantrolene), and clinically significant depression and anxiety were assessed.

## Results

Out of 205 patients, 66 reported cognitive concerns, of which 20 completed cognitive testing (Table 1). There was no statistically significant difference in gender, age, or duration of illness in individuals included in this case series vs. the remainder of the cohort, or between those included in the case series vs. those who reported cognitive concerns but did not have cognitive testing (all *p* > 0.05). Three participants completed testing with a speech and language pathologist using the Repeatable Battery for the Assessment of Neuropsychological Status [RBANS; (20)], and 17 completed testing with a psychologist using a wide array of instruments (Supplementary Table 1). Our cohort was mostly female (*n* = 15, 75%), had a mean age at time of cognitive testing of 47.4 years (SD = 12.4), and mean duration of illness of 10.1 years (SD = 7.6). Most had anti-GAD65 antibodies (17/20, 75%), and classic SPS phenotype (15/20, 75%). Three (15%) had a history of seizures, none of which were intractable or poorly controlled. Common classes of medications prescribed included benzodiazepines (*n* = 14, 70%), antidepressants (*n* = 13, 65%), non-benzodiazepine muscle relaxants (*n* = 10, 50%), and opioids (*n* = 4, 20%). Nine out of eleven (82%) patients assessed for depression reported clinically significant symptoms, and 4 out of 9 (44%) patients assessed for anxiety reported clinically significant symptoms.

**Table 1:** Clinical and laboratory features of patients with stiff person syndrome spectrum disorders who received formal cognitive testing as part of routine clinical care for patient-reported cognitive changes.

**Patient number**	**Baseline characteristics**							**Cognitive testing results**	
	**Age at testing**	**Years with SPS^f^**	**SPS phenotypes**	**Anti GAD-65 titer**	**Relevant medical comorbidities**	**Psychiatric comorbidities^a^**	**Psychoactive and immune-based medications**	**Areas of impairment**	**Psychiatric symptoms ^g^**
1	59	2	GAD+SPS Cerebellar predominant	63,525 IU/mL	Vitiligo B12 deficiency Remote Intestinal Ca Remote Testicular Ca	None	Clonazepam	Processing speed Verbal phonemic fluency	GDS-15: 8/15 NPI-Q: agitation, depression, apathy, irritability, nighttime behaviors, appetitive changes
2	39	<1	GAD -SPS	39 U/mL ^e^	T2DM B12 deficiency Vit D deficiency Narcolepsy Small fiber neuropathy Mild OSA	Depression Anxiety PTSD ADHD	Oxymorphone Oxycodone Pregabalin Metaxalone Baclofen Clonazepam Alprazolam Armodafinil Certirizine	No areas of impairment	PHQ-9 = 15 (moderately severe depressive symptoms)
3	74	8	GAD+SPS Cerebellar predominant	6.3 U/mL	Coronary artery disease	Depression	IVIG Duloxetine	Verbal learning and recall Motor speed Executive function (Set-shifting) Processing speed Verbal phonemic fluency	BAI 8 (minimal anxiety) PHQ-9 = 0 (no symptoms)
4	22	2	GAD+SPS	30 U/mL	Hypothyroidism Sickle cell anemia Asthma CVA partial seizures	Generalized anxiety disorder ^b^ Major depressive disorder, recurrent, moderate ^b^ Adjustment disorder due to medical condition ^b^	Baclofen Diazepam Benzonatate Diphenhydramine	Executive functioning (inhibition) Attention Verbal phonemic fluency Verbal recall Motor speed	PAI: severe depressive symptoms
5	60	8	GAD-Possible SPS	Not available	B12 deficiency Vit D deficiency Ankylosing spondylitis Hypertension OSA	None	Clonazepam Methotrexate Bupropion Tramadol	Verbal recall	Not assessed
6	29	17	GAD+SPS	250 IU/mL	Hypothyroidism Primary Immune deficiency Orthostatic hypotension Crohn's disease Chiari Malformation	None	Adalimumab Tacrolimus Clonidine Duloxetine Modafinil Prednisone Topriamate	Verbal learning and recall Executive functioning (set shifting) Attention Verbal phonemic fluency Verbal semantic fluency Working memory	Not assessed
7	54	12	GAD+SPS	21,888 U/mL	SLE	Depression Anxiety	Baclofen Diazepam Clonazepam IVIG Duloxetine Buspirone Doxylamine Melatonin	Verbal phonemic fluency Visual recall Executive function (set shifting) Attention Processing speed	BDI: 29 (moderate depression) PAI: significant depression and anxiety
8	43	6	GAD+SPS Plus	25,000 U/mL	Insulin dependent diabetes Epilepsy, sickle cell trait, migraines	None	Clonazepam Cyclobenzaprine Lacosamide Levitracetam Oxycodone	Language (verbal and reading comprehension, naming, spelling) Visual learning and recall	PAI: significant anxiety
9	36	4	GAD+SPS	320 IU/mL	Neuropathy Migraine	Anxiety Depression PTSD	Baclofen IVIG Clonazepam Diazepam Gabapentin Paroxetine	Verbal phonemic fluency Verbal semantic fluency Verbal recall Motor speed	PAI: significant anxiety, depression, anxiety related to past trauma and stress
10	59	20	GAD-Possible SPS	Not available	Cervical stenosis Migraines	None	Carbamazepine Tizanidine	Verbal recall^c^	Not assessed
11	49	12	GAD+SPS	117 IU/mL	None	Major Depressive Disorder, recurrent ^b^	Baclofen Bupropion Buspirone Clonazepam Diazepam IVIG Rituximab	Processing speed Attention Verbal recall Visuospatial judgement	Not assessed
12	59	3	GAD+SPS	615 nmol/L	None	Paranoid schizophrenia^b^	Fluoxetine Levitracitam Diazepam Olanzapine Memantine	Verbal learning and recall ^c^ Visual learning and recall Language (expression) Verbal phonemic fluency Motor speed Executive function (inhibition) Processing speed	BDI and BAI within normal limits (score not reported)
13	49	24	GAD+SPS	207,650 U/mL	Diabetes Mellitus Epilepsy (s/p temporal lobectomy) Hypothyroidism Pernicious anemia SLE	None	Baclofen Diazepam Lacosamide Levetiracetam Pregabalin	Processing speed Executive function (Set shifting) Verbal learning and recall Language (naming) Verbal phonemic fluency	PHQ-9 = 7 (mild depression) GAD-7 = 12 (moderate anxiety)
14	54	22	GAD+SPS	6.6 U/mL	Insomnia Postural orthostatic tachycardia syndrome Migraines	Generalized anxiety disorder^b^ Major depressive disorder^b^	Doxepin SCIG Pregabalin	Processing speed^c^ Verbal semantic fluency	PHQ-9 = 27 (severe depression) GAD-7 = 19 (severe anxiety)
15	45	9	GAD+SPS	213 IU/mL	Tublerculosis (1 yo) Coronary artery disease Dyslipidemia HTN Hypothyroidism	None	Clonazepam Diazepam Hydralazine IVIG	Verbal recall Verbal semantic fluency Executive functioning (Set-shifting) Processing speed Motor speed	Not assessed
16	45	23	GAD+SPS	174.2 U/mL	Anemia Anticardiolipin antibody positive T1DM Hepatitis Rheumatoid arthritis SLE	Major depressive disorder Anxiety	IVIG Baclofen Clonazepam Escitalopram Prednisone	Attention^d^ Verbal learning and recall Visual learning and recall	Not assessed
17	41	9	GAD+SPS	174.2 U/mL	Dysautonomia Idiopathic small fiber sensory neuropathy T1DM	None	Baclofen Clonazepam Diazepam Pregabalin Modafinil Oxycodone Roxicodone Paroxetine IVIG Tizanidine	Verbal phonemic fluency Motor speed	GAD-7 = 1 PHQ-9 = 19
18	41	3	GAD+SPS	53,650 U/mL	Anemia (iron deficiency) Eczema Asthma Seizures	Anxiety	Baclofen Diazepam Mirtazapine	Attention^d^ Visuospatial skills Verbal phonemic fluency Verbal learning and recall	Not assessed
19	33	7	GAD+SPS	34 IU/mL	Seizures Ataxia Nystagmus	None	Baclofen Escitalopram IVIG Levetiracetam Rituximab	Attention^d^ Verbal phonemic fluency Verbal recall Visual recall Visuospatial skills	Not assessed
20	56	1	GAD+SPS	Not available	SLE Psoriatic arthritis T1DM B12 deficiency Autoimmune thyroiditis Hypertension Sjogren's syndrome	Depression	Amlodipine Apremilast Diazepam Escitalopram Estradiol Clonazepam	Attention Verbal phonemic fluency Verbal recall Motor speed	Not assessed

a *Psychiatric diagnoses are based only on patient report unless noted otherwise*;

b *psychiatric diagnoses confirmed by psychiatric or psychologist notes*;

c *no raw score or percentiles from neuropsychological batteries were reported for these patients. Domains of impairment were only based on testing interpretation summary*;

d *only results of Repeatable Battery for Assessment of Neuropsychological Status (RBANS) completed by a Speech and Language Pathologist available*;

e *While this patient had one positive anti-GAD65 antibody test, at the time closest to neuropsychological testing, prior and subsequent tests have been negative, thus categorization of phenotype is GAD-*;

f *duration of illness calculated from time of symptom onset to time of cognitive testing*;

g *Psychiatric symptoms were considered clinically significant based on previously established cut-offs of >9 for the Generalized Anxiety Disorder Assessment (GAD-7) (14) and >4 for the Patient Health Questionnaire-9 (PHQ-9) (15), >9 for Beck Depression Inventory (BDI) (16), >7 for the Beck Anxiety Inventory (BAI) (17). For individuals whose psychiatric symptoms were only assessed using the PAI, clinically significant symptoms were identified based on interpretation in the neuropsychiatric report. GAD -, anti-glutamic acid decarboxylase antibody negative; GAD +, anti-glutamic acid decarboxylase antibody positive; SLE, systemic lupus erythematosus; T1DM, type 1 diabetes; OSA, Obstructive Sleep Apnea; PTSD, Post-traumatic stress disorder; ADHD, Attention Deficit Hyperactivity Disorder; CVA, Cerebrovascular accident; Ca, Cancer; s/p, status-post; IVIG= Intravenous immunoglobulin; SCIG= Subcutaneous immunoglobulin; NPI-Q, Neuropsychiatric Inventory – Questionnaire (18); PHQ-9, Patient health questionnaire-9 (15); GAD-7, Generalized anxiety disorder-7 (14); PAI, Personality assessment inventory (19); BDI, Beck depression inventory (16); BAI, Beck anxiety inventory (17)*.

Of the 20 patients who completed cognitive testing, 19 performed in the “impaired” range in at least one cognitive domain. The most common domains of impairment were verbal learning and recall memory (*n* = 14, 70%), verbal fluency (*n* = 11, 55%), processing speed (*n* = 8, 40%), attention (*n* = 8, 40%), motor speed (*n* = 7, 35%), semantic verbal fluency (*n* = 6, 30%), visual learning and recall memory (*n* = 5, 25%), set-shifting (*n* = 5, 25%), inhibition control (*n* = 3, 15%), and visuospatial processing (*n* = 3, 15%).

**Figure 1:**
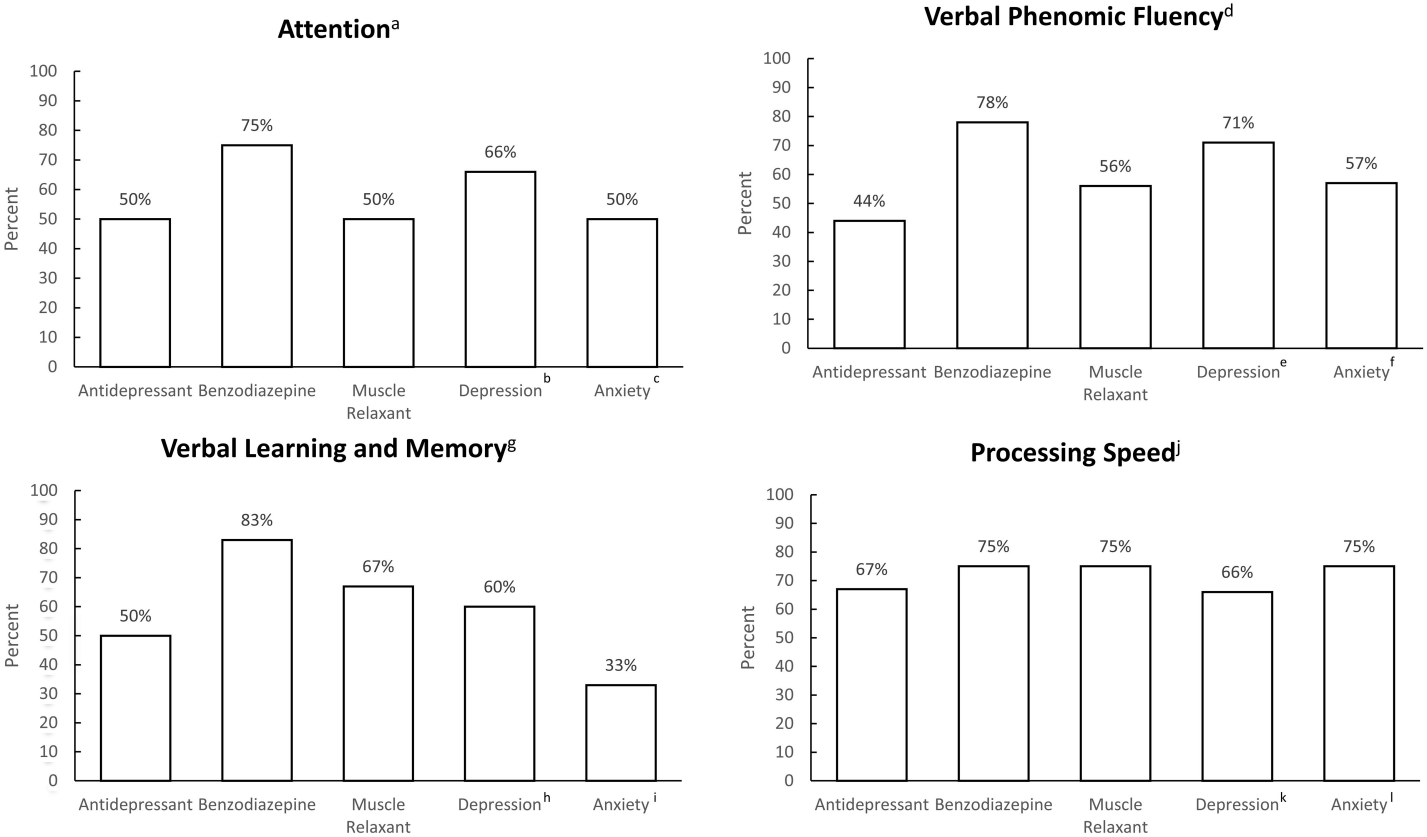
Frequency of antidepressant use, benzodiazepine use, non-benzodiazepine muscle relaxant use, and clinically significant depression and anxiety symptoms, grouped by most commonly impaired cognitive domains. ^a^*n* = 8, ^b^*n* = 3, ^c^*n* = 2; ^d^*n* = 11, ^e^*n* = 7, ^f^*n* = 7; ^g^*n* = 14, ^h^*n* = 11, ^i^*n* = 6; ^j^*n* = 8, ^k^*n* = 6; ^l^*n* = 4.

Patterns of medication use and clinically significant depressive and anxiety symptoms are described in Figure 1.

## Discussion

To our knowledge, this is the first detailed examination of cognitive and mood profiles in patients with SPSD who present with cognitive concerns. The most common cognitive domains exhibiting impairment were verbal recall, processing speed, attention, and phonemic verbal fluency. Additionally, results suggest an overlap of cognitive impairment with use of SPSD medications and presence of mood and anxiety symptoms. Reduced GABA levels have been associated with anxiety and depression (21), as well as cognitive impairment in schizophrenia (22), multiple sclerosis (23), and Alzheimer's disease (24). Metabolic abnormalities in the frontal cortex, temporal cortex, thalamus, and cingulate cortex (25) have been reported in classic SPS, regions that have previously been associated with psychiatric symptoms in cognitive disorders (26). Thus, there is a biological plausibility that cognitive impairment and mood and anxiety disorders are intrinsic to the disease process.

Our results expand on previously published work by Budhram et al. (11). Though cognitive findings specific to SPS phenotype were not reported separately, they found that 18% (*n* = 38) of their cohort with various anti-GAD65 associated neurological disorders had cognitive impairment as diagnosed by the Kokmen short test of mental status (11, 27). Consistent with our findings, the predominant cognitive domains impacted were verbal learning and recall memory (29/38, 76%), followed by working memory/attention (6/38, 16%), and verbal fluency/language processing (3/38, 8%). Similarly, another study of cognitive profiles in 21 patients with anti-GAD65-positive diabetes (without a co-existing neurological condition, severe psychiatric disorders or use of psychotropic medications) reported that performance on recall memory and phonemic verbal fluency tasks were significantly lower in anti-GAD65-positive individuals than in the control group (5). Psychiatric symptoms, however, were not evaluated in either study in relation to cognition.

Among the 20 patients included in our case series, 65% were prescribed antidepressants, and approximately half of those assessed for depression and anxiety reported clinically significant symptoms. This is consistent with prior studies (3, 28, 29), and a recent systematic review which found that the relative risk of psychiatric comorbidity in SPS was higher than that of the general population (7). Mood and anxiety disorders are associated with deficits in learning and memory, executive function, and attention—areas also impaired in SPSD and anti-GAD65 associated diseases (8, 9). Although the present findings are observational and cannot confirm causation, bidirectional pathways of mood and cognition have been established in longitudinal studies of other patient populations (30, 31).

Both benzodiazepines and muscle relaxants have been associated with increased risk of cognitive impairment (32–34). In particular, long-term benzodiazepine use has been associated with deficits in visuospatial processing, processing speed, and verbal learning (10). While we observed a high prevalence of these medications in individuals with cognitive impairment, future studies on the potential effects of these medications on cognition in SPSD are needed to establish causality. At a minimum, there should be increased consideration for their long-term use given the potentially harmful effects.

These findings should be interpreted within the context of their limitations. This was a convenience, retrospective sample of individuals who had completed cognitive testing following referral based on reported cognitive concerns. Testing was conducted at different sites and by different providers, without standardization of test selection or interpretation. Moreover, as previously noted, certain medications that are used in SPSD can influence cognitive function. Despite the aforementioned limitations, our present findings contribute to the limited literature on cognitive and mood profiles in patients with SPSD by identifying common domains of cognitive impairment and potential overlap of cognitive impairment with mood symptoms and medication use.

In summary, assessment of cognitive impairment in SPSD should include testing of verbal learning and recall, phonemic verbal fluency, attention, and processing speed. Cognitive screening tools that examine these domains, such as the Montreal Cognitive Test (MoCA), could be used in the clinical setting to help identify patients who may need additional cognitive evaluation. Psychiatric symptoms and use of medications that may affect cognition are common, and should be considered when evaluating cognitive impairment in this population. Further studies are needed to replicate these findings using longitudinal prospective study designs with consistent cognitive assessment tools and interpretive standards to further clarify the scope of neuropsychiatric disturbance in SPSD and their underlying mechanisms.

## Data Availability Statement

The raw data supporting the conclusions of this article will be made available by the authors, without undue reservation.

## Ethics Statement

The studies involving human participants were reviewed and approved by Johns Hopkins Institutional Review Board. The patients/participants provided their written informed consent to participate in this study.

## Author Contributions

CC: conceptualization of the study, data analysis, data interpretation, drafting, and revision of manuscript. DP, YW, and DO: data acquisition, data interpretation, and revision of manuscript. AH: data interpretation and revision of manuscript. SN: conceptualization of the study, data acquisition, data interpretation, study supervision, and revision of manuscript. All authors contributed to the article and approved the submitted version.

## Conflict of Interest

The authors declare that the research was conducted in the absence of any commercial or financial relationships that could be construed as a potential conflict of interest.

## Publisher's Note

All claims expressed in this article are solely those of the authors and do not necessarily represent those of their affiliated organizations, or those of the publisher, the editors and the reviewers. Any product that may be evaluated in this article, or claim that may be made by its manufacturer, is not guaranteed or endorsed by the publisher.
