# Supplementary material for: Cognitive and Mood Profiles Among Patients With Stiff Person Syndrome Spectrum Disorders
Source: Front Neurol. 2022 May 27;13:865462. doi: 10.3389/fneur.2022.865462 (PMC9184450; doi:10.3389/fneur.2022.865462)
Supplement: Supplementary file 1 [file Table_1.DOCX]

Supplementary table 1. Neuropsychological test battery used formal cognitive testing, performed by either a licensed psychologist or a speech and language pathologist, as part of routine clinical care for patient-reported cognitive changes.

| Patient number | Neuropsychologist tests |
| --- | --- |
| 1 | Hopkins adult reading test  Salthouse perceptual comparison test  Digit span  Brief test of attention  Boston naming test 30  Brief visuospatial memory test - revised  Rey complex figure test  Hopkins verbal learning test - revised  Wechsler Memory Scale IV: logical memory  Porteus maze test  Delis-Kaplan executive function system: tower test  Delis-Kaplan executive function system: color-word test  Delis-Kaplan executive function system: trail making test  Calibrated ideational fluency assessment  Neuropsychological assessment battery judgement  Grip strength |
| 2 | Mini mental state exam  Wechsler memory scale III  Wechsler adult intelligence scale III  Rey-Osterreith complex figure  Rey auditory verbal learning test  Boston naming test  Controlled oral word association  Boston diagnostic aphasia examination  Grooved pegboard test  Trail making test A  Trail making test B  Stroop test |
| 3 | Wide range achievement test 4  Judgement of line orientation  California verbal learning test II  Brief visuospatial memory test  Symbol-digit modalities test  Trail making test A  Trail making test B  Wechsler Memory Scale IV: digit span  Letter fluency  Delis-Kaplan executive function system: tower test  Reliable digit span  California verbal learning test II |
| 4 | Wechsler adult intelligence scale IV  Wide range achievement test 4  Neuropsychological assessment battery  Stroop test  Delis-Kaplan executive function system: symbol digit coding  Delis-Kaplan executive function system: shifting attention test  Delis-Kaplan executive function system: reasoning test  Finger tapping test  Four part continuous performance test  Verbal memory test |
| 5 | Prefrontal symptoms inventory  Wechsler adult intelligence scale IV  Trail making test A  Trail making test B  Wechsler abbreviated scale intelligence II  Hopkins verbal learning test  Rey-Osterreith complex figure  Judgement of line orientation  Wisconsin card sorting test  Verbal fluency |
| 6 | Grooved pegboard test  Wechsler adult intelligence scale IV  Delis-Kaplan executive function system: trail making test  Wechsler adult intelligence scale IV  California verbal learning test III  Wechsler memory scale IV  Delis-Kaplan executive function system: verbal fluency  Wisconsin card sorting test |
| 7 | Wechsler adult intelligence scale IV  Mini mental status exam  Mattis dementia rating scale  Trail making test A  Trail making test B  California verbal learning test  Wisconsin card sorting test  Confrontation naming  Verbal fluency  Wechsler memory scale 4 |
| 8 | Wechsler adult intelligence scale III  Wide range achievement test 4  Boston naming test 30  Verbal fluency Bakker-Brandt naming test  Hopkins verbal learning test – revised  Brief visuospatial memory test – revised  Grooved pegboard test |
| 9 | Mini mental state exam  Hopkins adult reading test  Trail making test A  Trail making test B  Modified Wisconsin sorting test  Digit Span  Wechsler memory scale IV  Verbal fluency  Clock drawing  Boston naming test 30  Brief visuospatial memory test - revised  California verbal learning test II  Wechsler memory scale IV  Grooved pegboard test  Grip Strength |
| 10 | Single word reading  Confrontation naming  Verbal fluency  Grip strength  Specific test battery used not described for: auditory attention, divided attention, sequencing ability, story memory, recognition for stories, visual learning, verbal learning verbal abstract, novel problem solving |
| 11 | Verbal fluency  Grip strength  Confrontation naming  Specific test battery not described for: spatial judgement, verbal repetition, single word reading, confrontation naming, auditory attention, repetition task, verbal learning, short recall, delayed recall |
| 12 | Wechsler adult intelligence scale IV  Speeded visuomotor number sequencing  Speeded color naming  Basic visuospatial figure copy  Visuoconstruction with blocks  Clock drawing and copy  Irregular word reading  Vocabulary knowledge  Confrontation naming  Verbal fluency  Verbal list learning  Visuospatial figure recall and recognition discrimination  Visuomotor set shifting  Response inhibition  Problem solving  Fine motor speed |
| 13 | Hopkins verbal learning test – revised  Trail making test A  Trail making test B  Stroop test  Weschler adult intelligence scale IV digit span  Boston naming test  Verbal fluency  Reliable digit span  Rey 15-item test |
| 14 | Judgement of line orientation  California verbal learning test 3  Brief visuospatial memory test  Trial making test A  Trail making test B  Symbol digit modalities test  Wechsler adult intelligence scale IV: digit span  Stroop test  Boston naming test  Reliable digit span  Rey 15-item test |
| 15 | Wide range achievement test 4  Brief visuospatial memory test  Hopkins verbal learning test  Symbol digit modalities test  Grooved pegboard test  Trail making test A  Trail making test B  Wechsler adult intelligence scale III: digit span  Verbal fluency  Wisconsin sorting test |
| 16 | Repeatable battery for the assessment of neuropsychological status |
| 17 | Grooved pegboard test  Boston naming test  Rey-Osterrieth complex figure  California verbal learning test II  Symbol digit modalities test  Brief visuospatial memory test  Wechsler adult intelligence scale IV  Verbal fluency  Wisconsin sorting test  Reliable digit span |
| 18 | Repeatable battery for the assessment of neuropsychological status |
| 19 | Repeatable battery for the assessment of neuropsychological status |
| 20 | Grooved pegboard test  Trail making test A  Wechsler adult intelligence scale IV: digit span  Wechsler adult intelligence scale IV: coding  Stroop test  Boston naming test  Wechsler adult intelligence scale III: vocabulary  Wechsler adult intelligence scale III: block design  Judgement of line orientation  Brief visuospatial memory test - revised  California verbal learning test II  Wechsler memory scale IV: logical memory |
